# Study on Electrical Behavior of TiO_2_ and ZnO Nanostructures: Resistive Switching and State Retention Under Pressure and Temperature Conditions

**DOI:** 10.3390/nano16181181

**Published:** 2026-09-18

**Authors:** Cristian E. Patiño, Daniel E. Nuñez, Y. Porras Ramírez, Jorge A. Calderón, Heiddy P. Quiroz, A. Dussan

**Affiliations:** 1 Grupo de Materiales Nanoestructurados y sus Aplicaciones, Departamento de Física, Universidad Nacional de Colombia—Bogotá, Cra. 30 No. 45-03 Edificio 404 Yu Takeuchi Lab. 121C/121B-1 Ciudad Universitaria, Bogotá 110001, Colombia; crpatinoa@unal.edu.co (C.E.P.); dnunezc@unal.edu.co (D.E.N.); yporrasr@unal.edu.co (Y.P.R.); jcalderonc5@ucentral.edu.co (J.A.C.); hpquirozg@unal.edu.co (H.P.Q.); 2 Grupo de Investigación en Nanotecnología, Bioingeniería y Transferencia Tecnológica, Cluster in Convergent Sciences and Technologies, Universidad Central, Bogotá 110311, Colombia

**Keywords:** oxide memristors, endurance performance, stability, HRS

## Abstract

TiO_2_ nanotubes and ZnO thin films were investigated as oxide-based memristive systems for resistive switching and state-endurance applications under variable environmental conditions. TiO_2_ nanotubes were synthesized by electrochemical anodization, while ZnO thin films were deposited on Ti substrates by DC magnetron sputtering. Structural, chemical, and morphological properties were examined by Raman spectroscopy, X-ray diffraction, X-ray photoelectron spectroscopy, and scanning electron microscopy. TiO_2_ exhibited a vertically aligned nanotubular morphology, whereas ZnO showed a granular thin-film surface. Electrical characterization was performed using Au top electrodes and Ti as the bottom electrode under atmospheric pressure and high-vacuum conditions, with temperature varied from 353 K down to 77 K. Both materials exhibited hysteretic current–voltage behavior associated with resistive switching, although their response was strongly influenced by morphology, defect distribution, and environmental conditions. TiO_2_ nanotubes showed stable high- and low-resistance states, with an ON/OFF ratio of approximately 4.65, indicating robust state endurance. The observed behavior was attributed to oxygen-vacancy-mediated transport, filament stabilization, and interface effects. These results highlight the relevance of comparing TiO_2_ and ZnO nanostructures for identifying oxide systems capable of maintaining resistive states under temperature and pressure variations, supporting their potential for low-power non-volatile memory applications.

## 1. Introduction

Since their conceptualization in 1971 by Leon Chua [1], memristors have been a subject of sustained interest in modern electronic materials research. However, the consolidation of their experimental realization required several decades: it was not until 2008 that a first system faithfully reproducing the hysteresis behavior in current–voltage curves of TiO_2_ thin films was developed [2]. Following the experimental confirmation of this technology’s feasibility, research on this class of electronic elements grew considerably, focusing primarily on new materials and applications. Among the most prominent candidates in this domain is ZnO, with direct application in information storage systems such as resistive random-access memories (RRAM) [3].

Beyond TiO_2_ and ZnO, other transition-metal oxides have also attracted interest as nanostructured materials for electronic and memristive device architectures. In particular, electrochemical anodization of Al/Nb bilayer systems enables the formation of defect-containing niobium oxide (NbO_x_) nanocolumn arrays with controlled column-like morphologies and different niobium oxide phases. Such anodically formed architectures broaden the range of oxide nanostructures that can be considered for the development of resistive electronic devices [4].

Within this framework, metal oxides occupy a central role, owing both to the reproducibility of their switching behaviors and to their accessible, low-cost fabrication techniques, positioning them as promising candidates for next-generation electronic devices, including energy-efficient memory storage and computing applications [5]. TiO_2_ is recognized for its high structural stability and for exhibiting a resistive switching (RS) mechanism frequently based on the formation of phases that act as highly efficient conductive filaments. ZnO, in turn, offers distinctive advantages in terms of optical transparency, biocompatibility, and morphological versatility, enabling its integration into flexible electronics and biomedical devices [6,7].

Understanding of the mechanisms governing resistive switching has advanced significantly, enabling the classification of RRAM devices according to the nature of their underlying interactions. Those in which metallic cation migration occurs through a thin solid electrolyte film are termed electrochemical metallization memories (ECM); those exhibiting stoichiometry changes induced by temperature variations driven by current flow are classified as thermochemical memories (TCM); and those based on oxides mediated by oxygen anion migration and cation valence changes are identified as valence change memories (VCM). These categories account for the switching behavior and conductive channel formation in the vast majority of devices currently under development [8,9].

In low-power applications, however, alternative mechanisms have been proposed, suggesting that charge transport arises from carrier redistribution driven by the presence of oxygen vacancies acting as impurity centers [10,11]. Furthermore, the growing demand for memristive materials has given rise to a new area of study concerning the optimization of information retention [12], the analysis of the influence of synthesis technique [6,13], energy consumption, and the effect of environmental factors such as humidity and oxygen presence [14,15]. Temperature, geometry, and resistivity play a critical role in resistive switching due to Joule heating effects within the conductive filament [16]. Although thermal effects have been explored in isolation, device behavior under variable pressure conditions—particularly in high-vacuum environments—remains an open area of investigation [17,18].

The main objective of this work is to systematically investigate and compare the electrical behavior of TiO_2_ nanotubes and ZnO thin films as oxide-based memristive systems under different pressure and temperature conditions. In particular, we aim to evaluate how these environmental conditions affect their resistive switching characteristics and the stability of their high- and low-resistance states. By comparing the response of the two oxide systems, this study seeks to identify the influence of their structural and defect-related characteristics on switching behavior and stability of the HRS and LRS under varying environmental conditions. The results provide insights into the potential of TiO_2_ and ZnO nanostructures for non-volatile memory applications operating under variable pressure and temperature conditions.

Previous studies, including our earlier works, have investigated resistive switching in TiO_2_- and ZnO-based structures and have established the important role of composition, oxygen-related defects, and nanostructure in determining their electrical response [10,13]. Temperature-dependent transport and environmental effects on oxide-based resistive switching have also been reported [14,15,16,17,18,19]. However, these studies have predominantly addressed individual materials, compositional modifications, or specific environmental variables, and therefore do not establish how structurally dissimilar oxide systems respond comparatively when subjected to variations in both temperature and pressure. In particular, a direct comparison between an ordered nanotubular architecture and a granular thin film morphology under high vacuum and extended temperature conditions can provide information on whether morphological and defect-related differences translate into different levels of environmental robustness of the resistive states.

## 2. Materials and Methods

### 2.1. Synthesis of ZnO Thin Films

Ti substrates (99.99% purity), with dimensions of 1.2 cm× 3.3 cm and a thickness of 50 µm , were cleaned with Alconox, Merk, Darmstadt, Germany, distilled water, and deionized water, and then dried with gaseous $N_{2}$. Thin films of ZnO (99.99% target purity) (Plasmaterials, Inc., Livermore, CA, USA) were deposited by DC magnetron sputtering (Intercovamex, Ciudad de México, México) at a power of 100 W and a working pressure of 2.5 × 10^−2^ Torr for 10 min. Electrical contacts were deposited using vacuum metal evaporation (PVD system) with aluminum masks. Then, 0.150 g of Au was deposited at a working pressure of 3 × 10^−5^ Torr. For electrode deposition, the samples were heated to 473 K for 40 s to promote Au adhesion to the sample surface. The samples with contacts were followed by an annealing process at the same temperature for 2 h to improve adhesion.

### 2.2. Synthesis of TiO_2_ Nanotubes

TiO_2_ nanotubes were fabricated by electrochemical anodization at room temperature. Two Ti foils were used, one as the cathode and the other as the anode, immersed in a solution of 0.25 wt% ammonium fluoride (NH_4_F) (Merck KGaA, Darmstadt, Germany), 2 wt% distilled water, and 97.75% ethylene glycol (Merck KGaA, Darmstadt, Germany), with constant agitation at 250 rpm. A square wave signal (alternating voltage) was applied, varying between 20 V for 5 min and 80 V for 1 min, with a total anodizing time of 12 min. Following the same contact deposition methodology, the electrode contacts were carried out.

### 2.3. Characterization Methods

Structural characteristics were analyzed using a Horiba Xplora Plus Raman microscope (HORIBA Group, Kyoto, Japan) with a laser interaction of 532 nm, in conjunction with a PANalytical Xpert Pro DY2690 X-ray diffractometer (PANalytical B. V., Almelo, The Netherlands) with a Cu-Ka source and 40 kV. Additionally, X-ray Photoelectron Spectroscopy (XPS) measurements were performed on an XPS/ISS surface characterization platform made by SPECS GmbH (SPECS Surface Nano Analysis GmbH, Berlin, Germany) with an Al-Ka X-ray source, operated at 200 W. Morphological properties were obtained using a Tescan Vega 3 scanning electron microscope (SEM) (Vortex Company-Bruker, Brno, Czech Republic). For environmental electrical behavior testing, the KeyFactor’s Probe Station environment module was used in vacuum ranges from 10^−5^ Torr and temperatures between 353 K and 77 K. I-V curves and robustness were evaluated with a Keithley Model 2460 (SEISA-Tektronix S.A. de C.V., Tizapán San Ángel, San Ángel, Mexico City, México) 6.5-digit resolution meter with copper wires (gauge), which were soldered to the Au contacts using silver paste and then to a tin-plated circuit board prepared for electrical characterization measurements.

## 3. Results

**Figure 1:**
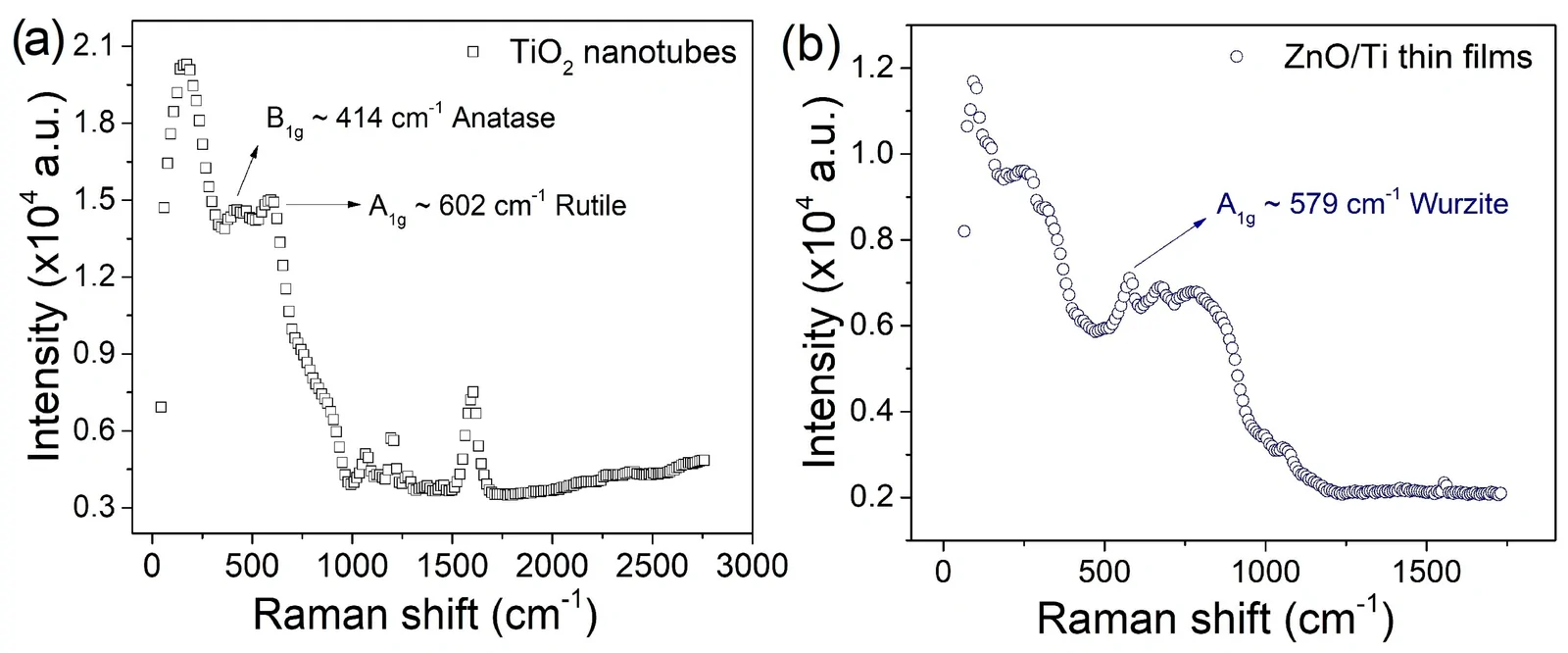
Raman spectra of (**a**) TiO_2_ nanotubes and (**b**) ZnO thin films on a Ti substrate.

Raman spectra of TiO_2_ nanotubes and ZnO thin films are presented in Figure 1. TiO_2_ has optical phonons at the Γ point $A_{1g}+A_{2g}+A_{2u}+{2B}_{1u}+B_{1g}+B_{2g}+E_{g}+3E_{u}$ where g represents Raman-active modes, u represents IR-active modes, and *E* represents degenerate modes [20]. Figure 1a shows Raman shifts associated with the anatase and rutile phases, with bands at 414 and 602 cm^−1^, respectively. The peak shift in both cases can be attributed to the amorphous contribution and defects in the TiO_2_ structure [21,22]. This behavior is associated with the synthesis process, which has resulted in the production of amorphous nanotubes [21,22].

ZnO crystallizes in the hexagonal wurtzite structure, belonging to the space group P6_3_ mc. Group-theoretical analysis predicts the optical phonons at the Γ point as A_1_ g + 2B_1_ g + Eg + 2Eu, where A_1_ and Eg are both Raman- and infrared-active, Eu modes are Raman-active only, and B_1_ modes are silent [23,24]. The A_1_ and E_1_ modes are further split into TO and LO components, while the E modes are doubly degenerate. In Figure 1b, the band at 579 cm^−1^ can be assigned to the longitudinal optical mode of ZnO, commonly associated with A_1_ g(LO) [11,12]. In contrast, the bands at 252 and 674 cm^−1^ are not typical first-order Raman modes of ideal wurtzite ZnO and are more likely related to second-order scattering processes, disorder-induced modes, or defect-associated contributions [25].

**Figure 2:**
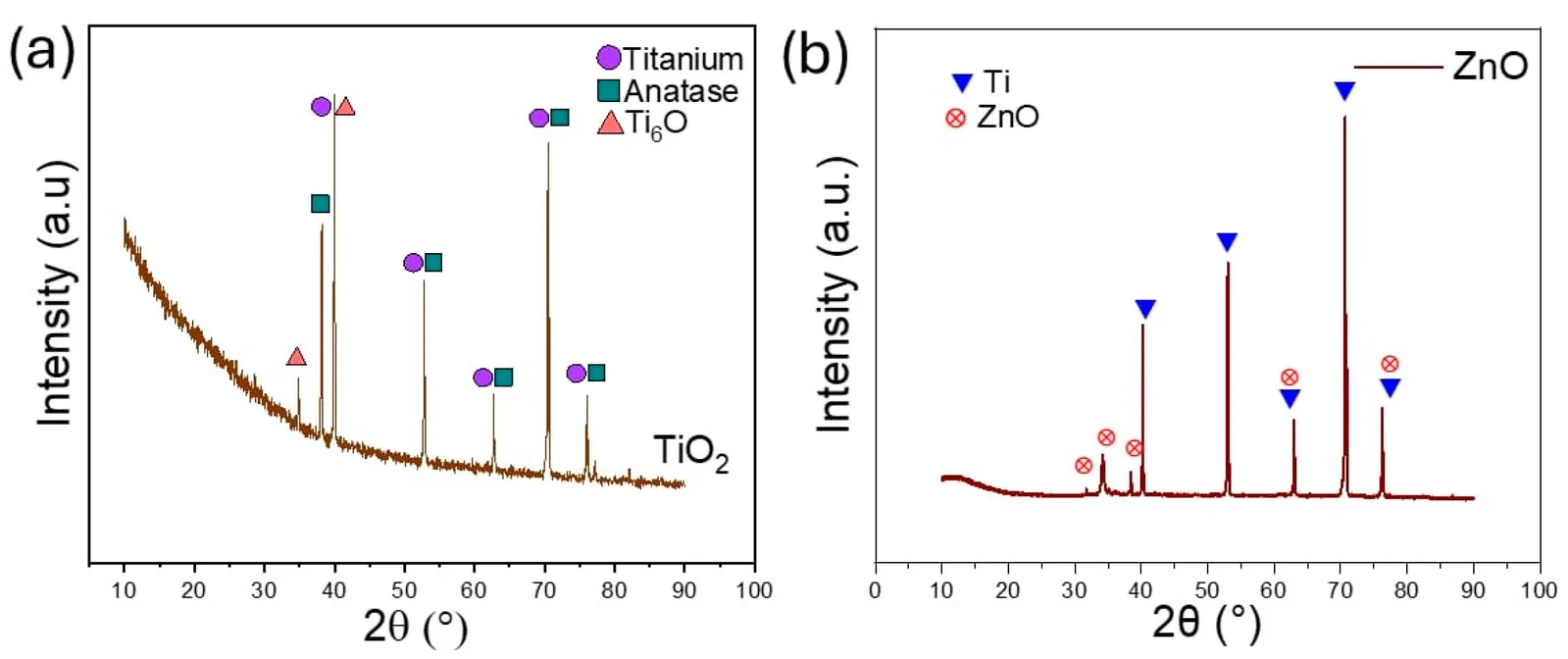
XRD pattern of (**a**) TiO_2_ nanotubes and (**b**) ZnO thin films on a Ti substrate.

Figure 2 shows the XRD patterns of the TiO_2_ nanotubes and ZnO thin films grown on Ti substrates. For the TiO_2_ nanotubes (Figure 2a), the diffraction pattern exhibits intense reflections originating from the metallic Ti substrate, together with peaks assigned to the anatase TiO_2_ phase. The presence of these reflections confirms the crystalline contribution of anatase within the anodized nanotubular layer. In addition, a broad diffuse background is observed, particularly at low diffraction angles, suggesting the coexistence of crystalline regions with structurally disordered or poorly crystallized material. Weak reflections assigned to the substoichiometric Ti_6_O phase are also observed. However, because some TiO_2_-related reflections overlap or occur close to the intense diffraction peaks of the Ti substrate, the phase assignment should be interpreted considering the contribution of the underlying metallic substrate.

The ZnO/Ti sample (Figure 2b) exhibits comparatively sharp diffraction peaks attributed to crystalline ZnO, together with intense reflections associated with the Ti substrate. Compared with the TiO_2_ nanotubular system, the ZnO film displays a lower diffuse background and more clearly defined ZnO-related reflections with wurtzite phase, indicating a significant crystalline contribution from the sputtered ZnO layer. The strong Ti reflections observed in both samples are expected because the X-ray penetration depth allows a substantial contribution from the underlying metallic substrate.

**Figure 3:**
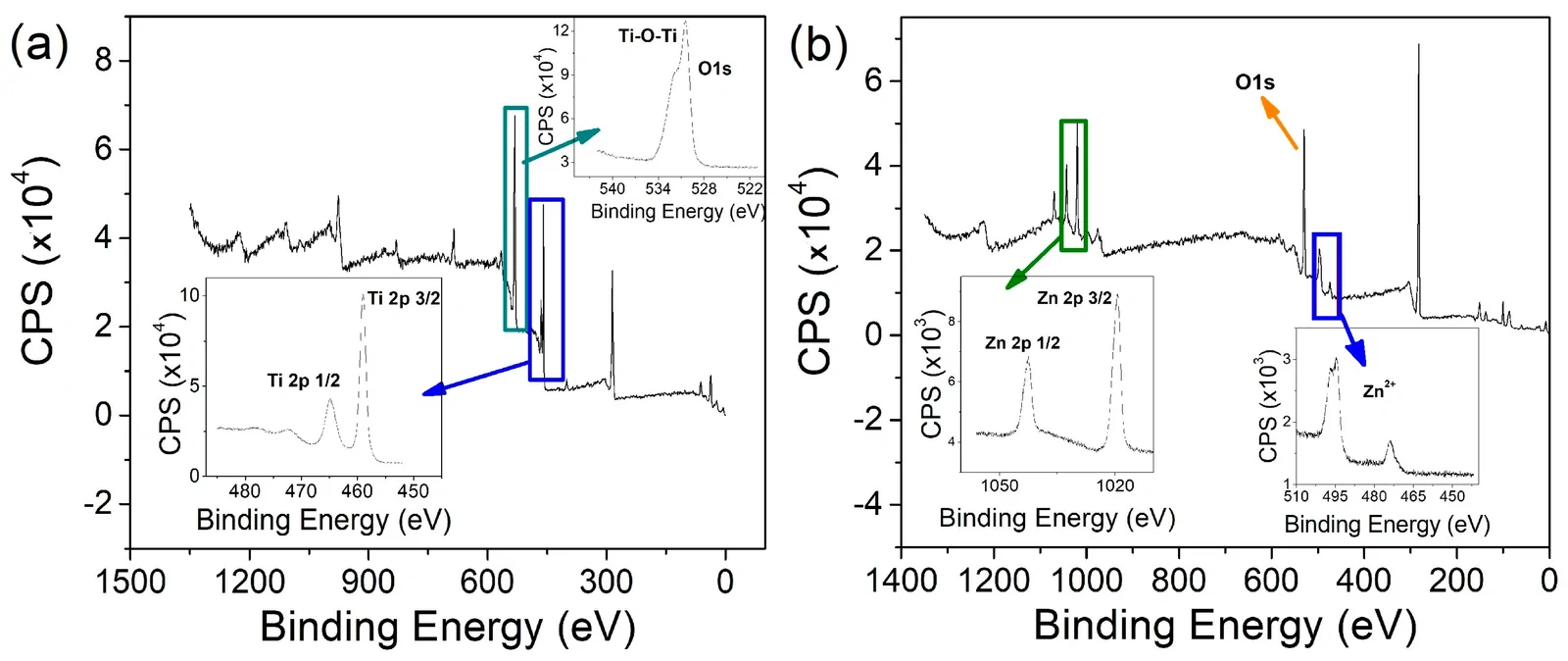
HR XPS spectra of (**a**) TiO_2_ nanotubes and (**b**) ZnO thin films. The inset figures represent the high-energy (green box in the high-resolution spectrum) and low-energy (blue box in the high-resolution spectrum) regions.

Figure 3 shows XPS spectra of the samples with HR spectra of Ti, Zn, and O identifications. The high-resolution Ti 2p XPS spectrum (see lower inset Figure 3a) exhibits two well-defined peaks located at binding energies of approximately 459 eV and 464.8 eV, corresponding to the Ti 2p_3_/_2_ and Ti 2p_1_/_2_ components, respectively [26]. The observed spin–orbit splitting of ~5.8 eV is consistent with the characteristic signature of Ti^4+^ in TiO_2_. The absence of additional features or shoulder peaks at lower binding energies indicates that titanium is predominantly in the Ti^4+^ oxidation state. The high-resolution O 1s spectrum exhibits a dominant peak centered at approximately 530.4 eV, which is attributed to lattice oxygen (O^2−^) bonded to Ti^4+^ in the TiO_2_ structure. A secondary component at higher binding energies (≈532.1 eV) is also observed, which is commonly associated with oxygen vacancies, surface hydroxyl groups, or defect-related oxygen species [27].

Depending on the number of trapped electrons, an oxygen vacancy may exist as an empty vacancy, a singly occupied vacancy, or a doubly occupied vacancy. In the terminology commonly used for oxide defect centers, the singly occupied and doubly occupied states are associated with F^+^- and F-type centers, respectively, and their electronic properties can differ substantially [28]. Other studies discussed the behavior of F-type centers in oxides and emphasized the distinction between one-electron and two-electron vacancy states, while more recent studies have shown that different vacancy-related defect populations can contribute differently to the optical and electronic response of oxide materials [29].

The high-resolution Zn 2p XPS spectrum exhibits two well-defined peaks centered at approximately 1019.4 eV and 1042.5 eV, corresponding to the Zn 2p_3_/_2_ and Zn 2p_1_/_2_ components, respectively. The observed spin–orbit splitting of about 23,1 eV is characteristic of Zn^2+^ species in ZnO [29]. This assignment is further supported by the Zn LMM Auger spectrum, whose line shape and kinetic energy position are consistent with oxidized zinc rather than metallic Zn. Taken together, the Zn 2p and Zn LMM results confirm that zinc is predominantly present in the Zn^2+^ oxidation state, as expected for ZnO [29].

**Figure 4:**
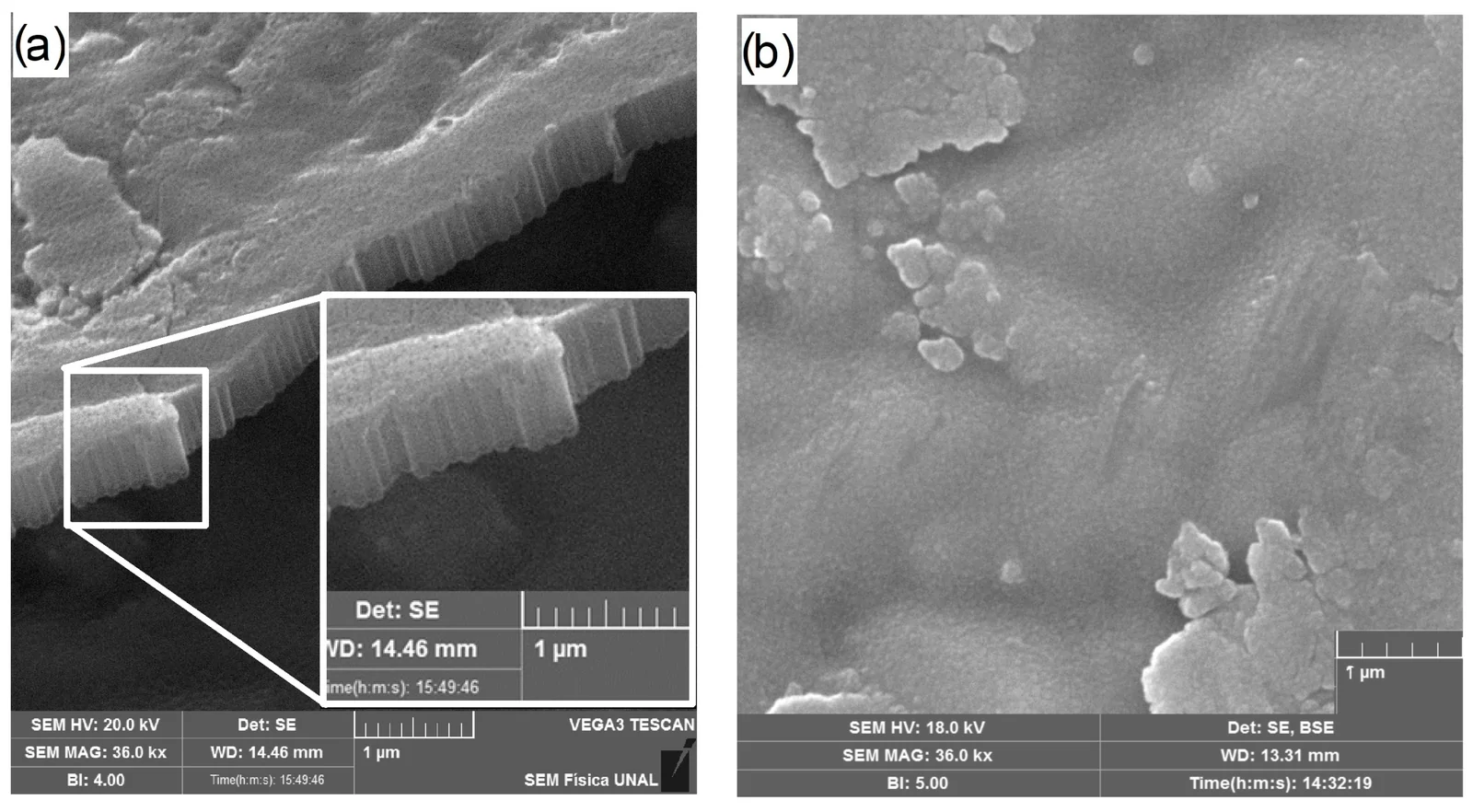
SEM micrographs of (**a**) TiO_2_ nanotubes and (**b**) ZnO thin films.

Topographic properties of the samples were investigated by scanning electron microscopy (SEM), as shown in Figure 4. The TiO_2_ nanotubes (Figure 4a) exhibit a well-defined tubular morphology, vertically aligned and uniformly distributed over the substrate. The nanotubes present an average length of ~518.9 ± 15.57 nm and a diameter of ~30.2 ± 3.62, forming a highly ordered array with relatively homogeneous dimensions. The cross-sectional view confirms the formation of densely packed nanotube walls, which are expected to favor directional charge transport along the tube axis.

In contrast, the ZnO thin films deposited on titanium foil (Figure 4b) display a granular surface morphology, characterized by the presence of agglomerated grains and irregular clusters distributed across the surface. Additionally, the formation of scale-like features can be observed, which are associated with the underlying substrate and the growth dynamics of the film.

**Figure 5:**
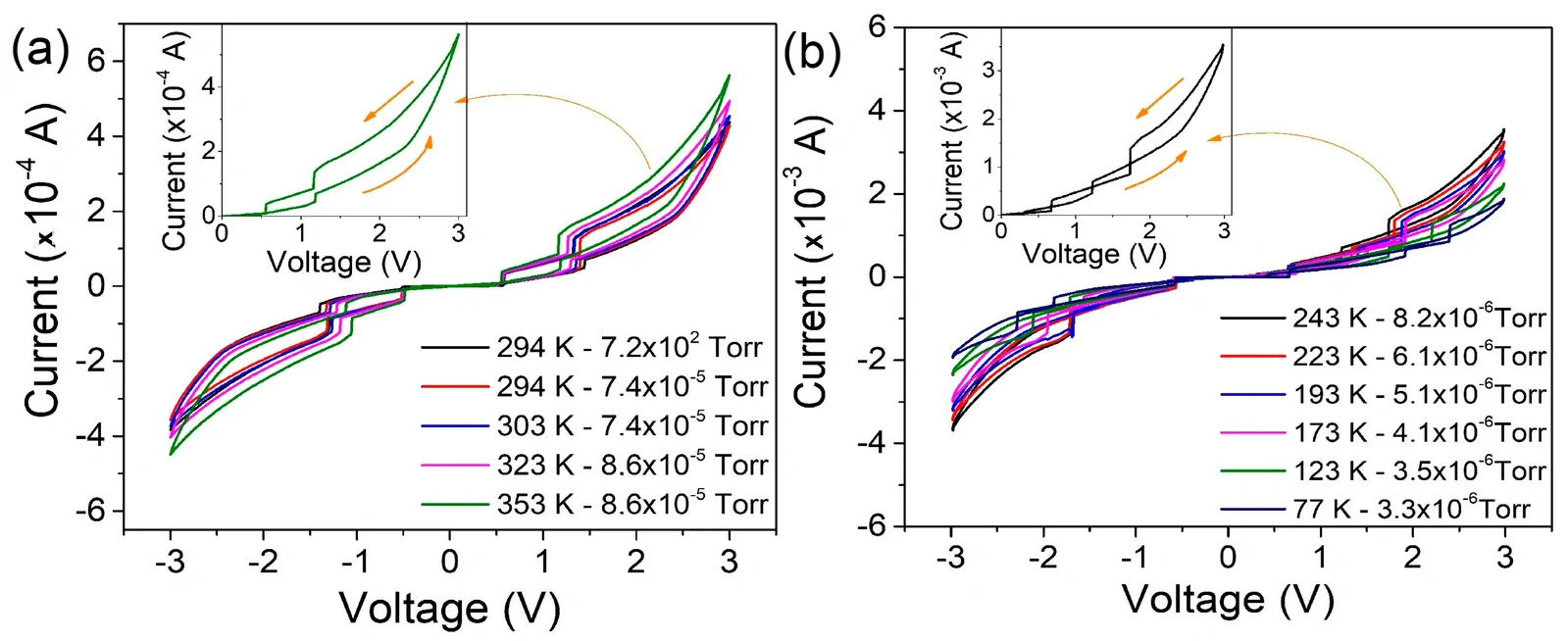
I–V curves of TiO_2_ nanotubes varying with (**a**) high temperature and (**b**) low temperature.

On the other hand, the electrical response of the TiO_2_ nanotube devices was evaluated under two different environmental conditions. First, measurements were carried out at room temperature and atmospheric pressure. Subsequently, the devices were characterized under high-vacuum conditions while varying the temperature from 353 K down to 77 K (Figure 5). The stability of the hysteretic I–V response and the evolution of the resistive states were investigated using TiO_2_ nanotubes with Au top electrodes (TE) and Ti foil as bottom electrodes (BE). Temperature-dependent I–V characteristics of TiO_2_ nanotubes were measured under high-vacuum conditions during (a) heating and (b) cooling. The pressure values indicated in the legend correspond to the residual chamber pressure recorded at each temperature and should not be interpreted as independently controlled pressure setpoints.

Figure 5a shows the I–V characteristics obtained under vacuum conditions while increasing temperature from room temperature up to 353 K. The I–V characteristics exhibit a temperature-dependent shift, accompanied by an increase in the current magnitude, particularly at 353 K. Nevertheless, the characteristic hysteretic response associated with resistive switching is maintained across the investigated temperature range, indicating that the switching behavior persists under thermal variations. This behavior suggests an enhancement of charge transport processes due to thermally activated carrier mobility and increased oxygen vacancy dynamics. At higher temperatures, the migration of ionic defects and local charge redistribution become energetically favored, facilitating conductive pathway formation and increasing the effective conductivity of the device.

In contrast, Figure 5b presents the I–V curves obtained at lower temperatures under high vacuum conditions. Despite cooling down to 77 K, the devices continue exhibiting clear hysteretic switching behavior, demonstrating remarkable stability of the memristive response. Nevertheless, a gradual reduction in current magnitude is observed as temperature decreases. Such behavior can be associated with reduced ionic mobility and suppressed defect migration at cryogenic temperatures, limiting conductive filament evolution. Despite these changes, the persistence of well-defined switching events indicates that the resistive states remain accessible even under strong thermal constraints.

Interestingly, the switching voltages exhibit only moderate variations over the explored temperature range, suggesting that the underlying transport mechanism possesses good thermal robustness. The preservation of the hysteretic response under both heating and cooling conditions indicates that the conductive channels formed within the TiO_2_ nanotubes remain sufficiently stable against external perturbations. These findings demonstrate that TiO_2_ nanotube devices exhibit reliable operation under extreme environmental conditions.

**Figure 6:**
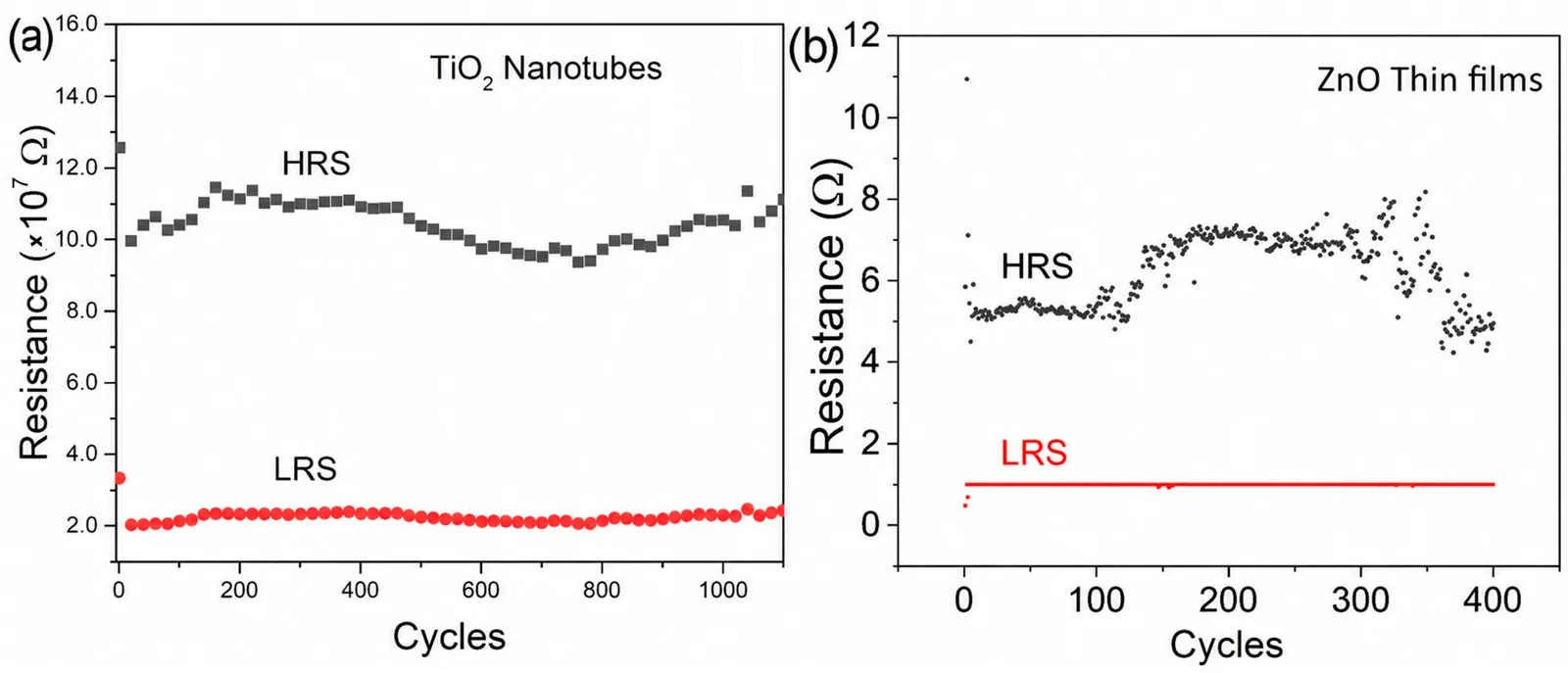
Endurance performance of (**a**) TiO_2_ nanotubes for 1200 cycles, and (**b**) ZnO thin films for 500 cycles, with consecutive voltage-sweep cycles from −3 to +3 V, measured at room temperature under $4.8\times 10^{2}$ Torr.

A similar trend is observed in the endurance test for TiO_2_ nanotubes (Figure 5b), where the conductive filament formation also occurs during the initial cycles, after which the switching behavior stabilizes. In this case, the LRS values remain relatively stable, while the HRS shows minor fluctuations, with an On/Off ratio of 4.65 (see Figure 6a).

Figure 6 presents the evolution of the high-resistance state (HRS) and low-resistance state (LRS) of the TiO_2_ nanotube device during 1200 and ZnO thin films during 500 consecutive switching cycles for both cases. A clear separation between both resistive states is maintained throughout the entire measurement, demonstrating the stability and reproducibility of the resistive switching process in TiO_2_ and ZnO.

The cycling endurance of the TiO_2_ nanotube device over 1200 consecutive complete voltage-sweep cycles from −3 to +3 V was measured. For each cycle, the HRS and LRS resistance values were extracted using the same read-voltage criterion, resulting in 1200 HRS and 1200 LRS values. The mean resistance values calculated over the complete cycling sequence allowed determining the value of the resistance-state ratio; these mean values are $\bar{R_{HRS}}/\bar{R_{LRS}}=4.65$. This value is used consistently throughout the manuscript to quantify the separation between the two resistance states. Nevertheless, cycling stability and temperature-dependent switching should not be interpreted as direct measurements of temporal data retention.

The mean HRS/LRS resistance ratio of approximately 4.65 provides a clearly distinguishable resistance window that remains stable throughout the investigated cycling sequence. Nevertheless, this ratio is relatively modest compared with the larger resistance windows generally desirable for conventional high-density non-volatile memory applications, where a larger separation between HRS and LRS improves the read margin and tolerance to electrical and device-to-device variability. The practical minimum resistance ratio, however, depends on the sensing scheme, device architecture, and intended application, and relatively low resistance ratios may remain functional in appropriately designed memory and in-memory-computing architectures.

The HRS remains within the range of approximately (9.4–11.5) × 10^7^ Ω, while the LRS fluctuates around (2.0–2.4) × 10^7^ Ω. Although small cycle-to-cycle variations are observed in both states, no significant degradation or progressive convergence between HRS and LRS was detected. The fluctuations are attributed to minor local rearrangements of defect-related conductive pathways and variations in charge trapping and detrapping processes within the oxide matrix. The average resistance ratio between HRS and LRS remains close to 4.65 during the entire cycling test, confirming the robustness of the memory window. These results demonstrate that TiO_2_ nanotubes possess excellent endurance characteristics and stability of the HRS and LRS, which are desirable features for non-volatile memory applications operating under repetitive switching conditions.

The cycling endurance behavior of the ZnO thin-film device is shown in Figure 6b. The ZnO device maintains distinguishable HRS and LRS over approximately 400 consecutive switching cycles. The LRS remains comparatively stable throughout the measurement, whereas the HRS exhibits greater cycle-to-cycle variability, with resistance values predominantly distributed between approximately 5 and 8 Ω after the initial cycles. Despite this variability, no progressive overlap between the two resistance states is observed within the investigated cycling range, indicating preservation of the resistive switching window. For ZnO thin films, the value of the resistance-state ratio is given by these mean values as $\bar{R_{HRS}}/\bar{R_{LRS}}=6.3$. The resistance window remained clearly distinguishable over the investigated cycling range.

**Figure 7:**
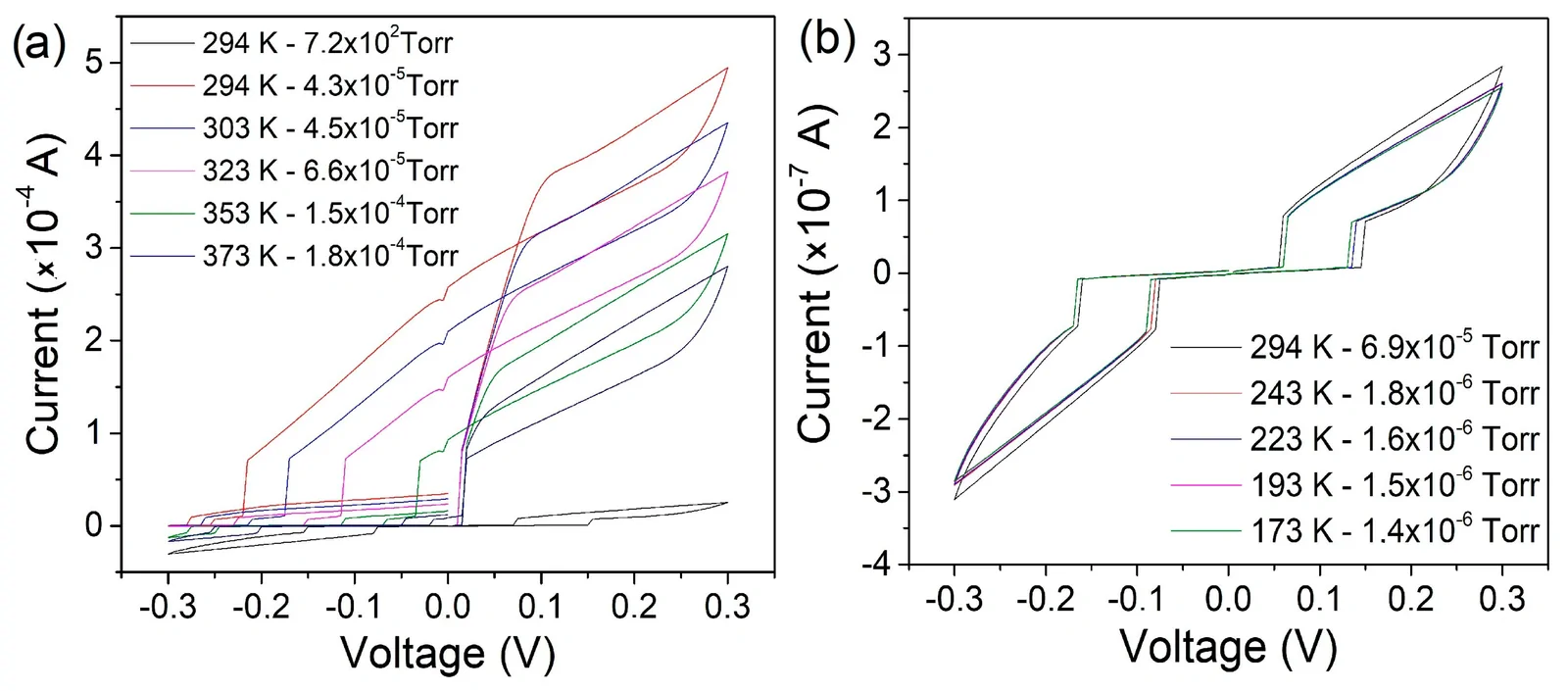
I-V curves of ZnO thin film varying with (**a**) high temperature and (**b**) low temperature.

For comparison, the electrical response of the ZnO thin films was evaluated under similar experimental conditions, with the temperature varied from 373 K to 172 K and the pressure also varied, as shown in Figure 7. Unlike TiO_2_ nanotubes, ZnO films exhibited a stronger dependence of the current magnitude on both temperature and pressure. Nevertheless, the hysteretic behavior remained observable throughout the explored temperature range, indicating the persistence of memory-related transport mechanisms. Although the current magnitude decreased substantially upon cooling, the qualitative hysteretic switching signature remained observable throughout the experimentally accessible ZnO temperature range.

Figure 7a shows the I–V characteristics measured under vacuum conditions at temperatures between 294 K and 373 K. A significant increase in current is observed when the temperature is increased from room temperature to 303 K, reaching the highest conductivity values within the explored range. This behavior suggests a thermally activated transport mechanism, where charge carrier mobility and defect-assisted conduction are enhanced at moderate temperatures. The presence of abrupt current transitions at specific voltages indicates discrete switching events, which may be associated with the activation of conductive pathways or charge trapping–detrapping processes within the ZnO matrix.

Figure 7b presents the I–V characteristics obtained at cryogenic temperatures under high-vacuum conditions. In contrast to the behavior observed in TiO_2_ nanotubes, the current decreases by approximately three orders of magnitude, from 10^−4^ A to 10^−7^ A, indicating a strong suppression of charge transport at low temperatures. Despite this reduction, the hysteretic response remains visible, demonstrating that the switching mechanism survives even when thermal activation processes are significantly reduced.

## 4. Discussion

The structural and spectroscopic analyses further suggest that defects play a fundamental role in the switching response. Raman measurements of TiO_2_ revealed the coexistence of anatase and rutile contributions accompanied by spectral shifts and broadening, indicating the presence of structural disorder and amorphous regions. Likewise, XPS analysis of the O 1 s signal evidenced contributions associated with oxygen-deficient environments and defect-related oxygen species. Oxygen vacancies are widely recognized as dominant active centers in valence change memory systems [21,22], acting as nucleation sites for conductive filament formation and charge transport channels. Their redistribution under electrical stress may promote reversible changes in local conductivity and therefore stabilize resistive switching [23]. Similar oxygen-defect-mediated conduction processes have been extensively described in TiO_2_ and ZnO-based RRAM devices.

Quantitative morphological analysis further highlights the structural differences between both oxide systems. The TiO_2_ nanotubes exhibit diameters below approximately ~30 nm and an average length of 518.9 ± 15.57 nm, whereas the ZnO film presents characteristic grain dimensions in the same order of magnitude (tens of nanometers) and a thickness of approximately 90 nm. Thus, although the characteristic lateral dimensions are comparable, the two systems differ substantially in their structural organization and active-layer thickness. The vertically aligned TiO_2_ nanotubes provide an ordered architecture, whereas the ZnO film contains a network of grains and grain boundaries that can act as preferential regions for defect-assisted charge transport. In addition, the thickness of the switching layer is known to influence resistive-switching parameters, including conductive-path formation and SET voltage, while the microstructure and defect distribution of sputtered ZnO films depend strongly on the growth conditions. Therefore, the different electrical responses observed here are likely associated with the combined influence of morphology, thickness, defect distribution, and fabrication route rather than with grain or nanotube diameter alone [30].

The electrical behavior observed in TiO_2_ nanotubes and ZnO thin films can be interpreted by considering differences in morphology, defect structure, and conductive mechanism established during synthesis. The SEM observations revealed that TiO_2_ develops an ordered nanotubular architecture, whereas ZnO exhibits a granular morphology composed of agglomerated surface features. Such structural differences are expected to strongly influence charge transport mechanisms and conductive filament formation. In nanotubular systems, charge carriers may preferentially propagate along vertically aligned “channels” defined by the nanotube walls, promoting directional transport and localized defect accumulation. In comparison, the granular nature of ZnO thin films introduces a larger density of grain boundaries and interfacial discontinuities, where charge trapping and scattering processes may occur. Similar effects have been reported in oxide-based resistive memories, where geometry and microstructure significantly influence filament formation and switching reproducibility [21,22,23,24,25,26,27,28,29,30,31,32].

The differences observed in resistive switching stability can also be associated with distinct conductive channel dynamics in both systems. In TiO_2_ nanotubes, the tubular geometry may facilitate the formation of distributed conductive pathways along the nanotube walls rather than abrupt localized filament rupture. Such transport behavior can favor switching reproducibility and improve stability of the HRS and LRS during repeated cycling. The endurance results demonstrated that the separation between HRS and LRS remained preserved over extended operation, indicating stable conductive path evolution. Small resistance fluctuations observed during cycling may be attributed to stochastic oxygen vacancy redistribution and subtle modifications of filament geometry. Similar cycle-to-cycle variations have frequently been reported in oxide memristive systems without significantly affecting functionality.

Environmental conditions are also expected to affect the electrical response through defect-mediated transport mechanisms. Previous studies have demonstrated that pressure, oxygen concentration, and temperature directly influence charge transport and conductive filament stability in oxide materials. Under vacuum conditions, surface adsorbates and oxygen-related interactions may be suppressed, modifying vacancy populations and charge trapping processes. Additionally, temperature variations can alter ionic mobility and Joule-heating-assisted filament evolution. Therefore, the comparative study performed under atmospheric and vacuum conditions provides additional insight into the robustness of the switching process under external perturbations in the TiO_2_ nanotubes and ZnO thin films. Such behavior is particularly relevant for practical applications involving harsh environments, flexible electronics, and low-power non-volatile memory technologies.

The preservation of hysteresis under both heating and cooling conditions suggests that the observed switching is not solely governed by thermally activated conduction but also involves defect-mediated memory effects. Oxygen vacancies, grain-boundary states, and localized charge trapping centers are likely to contribute to the switching process. The strong temperature dependence of the current indicates that ZnO exhibits a larger activation energy for charge transport than TiO_2_ nanotubes, making its electrical response more sensitive to environmental conditions.

A notable difference between both oxide systems is the magnitude of the temperature dependence. While TiO_2_ nanotubes preserve similar current levels over the explored temperature range, ZnO films experience a pronounced reduction in conductivity at cryogenic temperatures. This behavior may be attributed to the different conduction pathways available in each material. The vertically aligned nanotubular architecture of TiO_2_ facilitates the formation of stable defect-mediated conductive channels, whereas charge transport in ZnO is more strongly influenced by grain boundaries, surface states, and thermally activated carriers. Within the experimental conditions explored in this study, TiO_2_ nanotubes exhibit a comparatively more stable electrical response, whereas ZnO thin films show a stronger temperature dependence of the current. However, because the two systems differ in morphology, thickness, architecture, synthesis route, and temperature range, this comparison should be interpreted qualitatively rather than as evidence of an intrinsic superiority of one material over the other.

It should be emphasized that the TiO_2_ and ZnO devices were not designed as geometrically and experimentally matched structures. Therefore, the differences observed in their electrical behavior reflect the combined influence of material composition, morphology, defect distribution, device architecture, and measurement conditions. The present comparison is thus intended to identify qualitative trends in environmental response rather than to isolate the intrinsic contribution of a single parameter.

**Figure 8:**
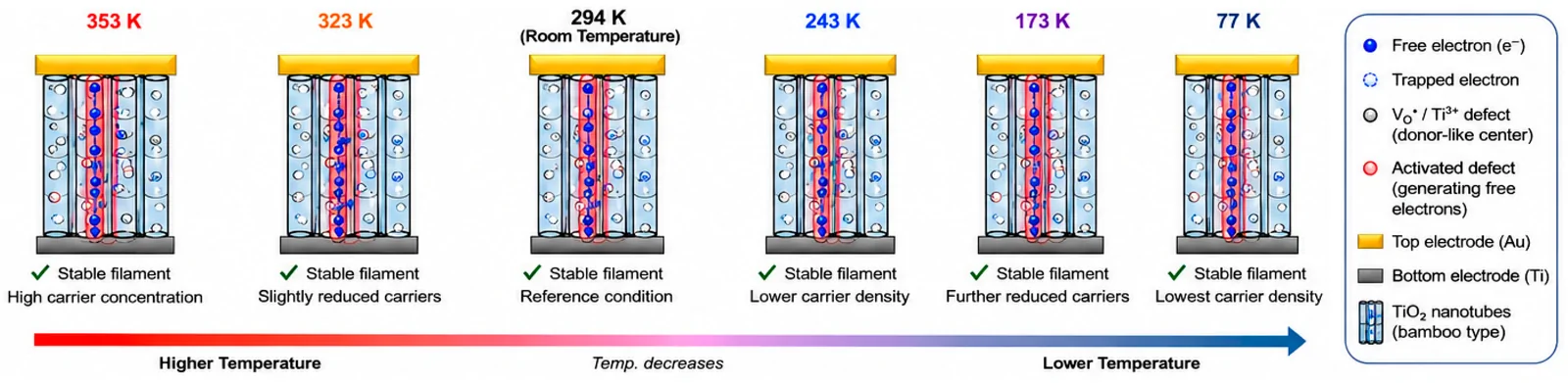
Conductive filament stability under temperature in high vacuum conditions. $V_{0}^{*}$ is associated to defects.

The electrical response observed in both TiO_2_ nanotubes and ZnO thin films can be interpreted in terms of defect-assisted electronic transport. In this scenario, the conductive filament is not formed by the long-range migration of oxygen ions but rather by the establishment of preferential electron percolation pathways through defect-rich regions distributed within the oxide matrix [23]. Oxygen vacancies, Ti^3+^ centers, and other structural defects act as donor-like states capable of releasing free electrons, thereby increasing the local carrier concentration and facilitating electronic conduction under an applied electric field (see Figure 8).

When a positive bias is applied, electrons injected from the electrodes preferentially occupy and connect neighboring defect states, progressively creating a continuous low-resistance conduction channel between both electrodes. The transition from the high-resistance state (HRS) to the low-resistance state (LRS) is therefore associated with the formation of an electronically connected defect network rather than with the physical growth of a metallic filament. During the RESET process, electron redistribution and trapping at defect sites partially disrupt this percolation pathway, restoring the high-resistance state while preserving the underlying defect structure.

The endurance measurements reveal different cycling-stability characteristics for the two oxide systems. TiO_2_ nanotubes exhibit comparatively stable HRS and LRS values with a well-preserved resistance window, whereas the ZnO thin film maintains two distinguishable resistance states but shows greater variability in the HRS. This behavior may be associated with the different structural organization of the active layers. In the granular ZnO film, grain boundaries and the spatial distribution of defects can contribute to cycle-to-cycle variations in the formation and redistribution of conductive paths, whereas the vertically organized TiO_2_ nanotubular architecture may provide comparatively more reproducible pathways for resistive switching. Nevertheless, because the devices differ in material, morphology, thickness, and fabrication route, the endurance differences cannot be attributed exclusively to morphology. The present results therefore demonstrate cycling stability in both systems within the investigated ranges, while indicating greater HRS variability in the ZnO device.

**Figure 9:**
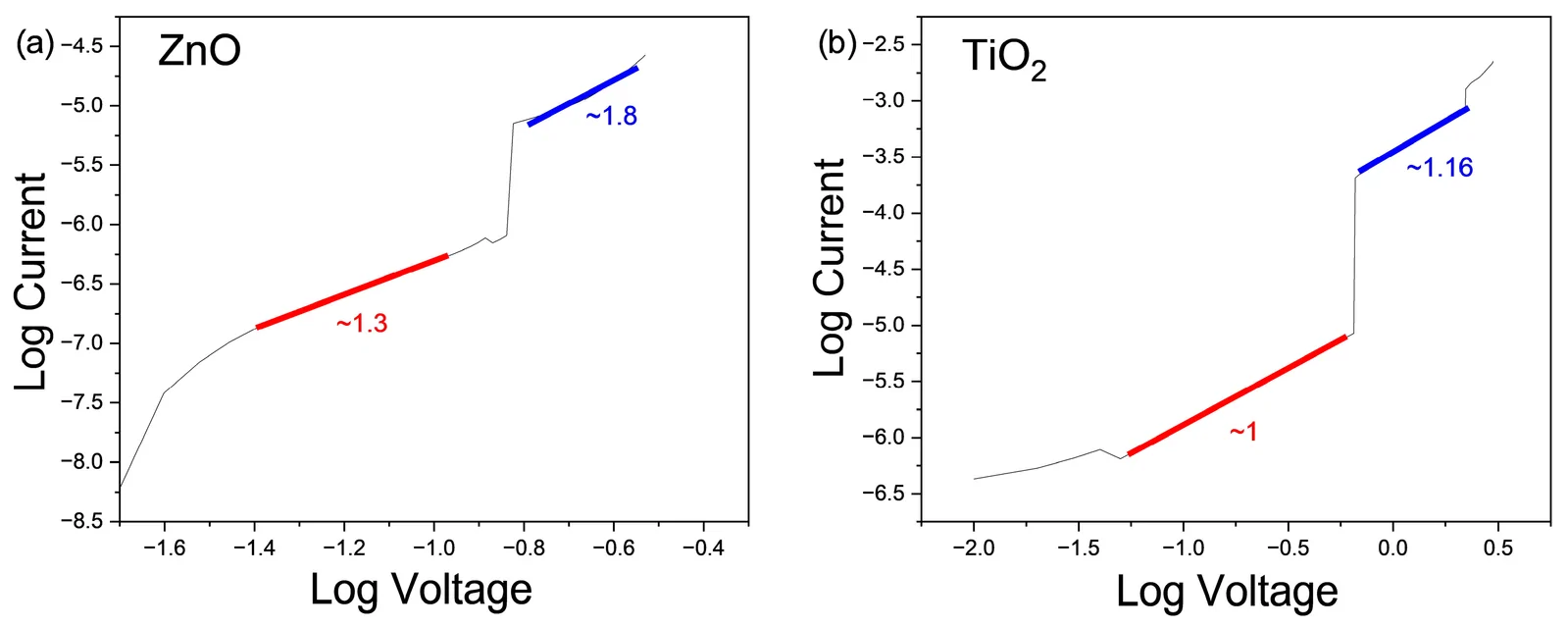
Log–log representation of I-V curves for (**a**) ZnO thin films and (**b**) TiO_2_ nanotubes. (Red color lower bias and blue color resistive-switching region).

To further investigate the charge-transport behavior associated with the resistive-switching response, the I–V characteristics were analyzed using the power-law relation $I\propto V^{m}$, which can be expressed in logarithmic form as log(I)=mlog(V)+C. Therefore, the slope m of the linear regions in the log–log representation provides information about changes in the dominant conduction regime. Figure 9 shows the corresponding analysis for the ZnO thin films and TiO_2_ nanotubes.

For ZnO (Figure 9a), two distinct regions are observed, with the power-law exponent increasing from approximately m=1.3 at lower bias to m=1.8 after approaching the resistive-switching region. The initial value, although slightly higher than the ideal Ohmic value of m=1, indicates a nearly linear voltage dependence with a moderate contribution from non-Ohmic processes. In contrast, the increase to m≈1.8 indicates a substantially stronger nonlinear contribution to charge transport. These exponents approach the quadratic dependence ($I\propto V^{2}$) commonly associated with trap-mediated or space-charge-influenced conduction. A similar evolution has been reported for ZnO-based resistive-switching structures, where a transition from an approximately linear regime (m≈1.8) to a nonlinear regime with m≈1.98 was observed prior to switching [33].

Nevertheless, the present value of m≈1.8 should not be interpreted as conclusive evidence of ideal space-charge-limited current (SCLC). Rather, it indicates that the ZnO conduction becomes increasingly nonlinear with increasing bias and is consistent with an enhanced contribution from localized states, carrier trapping, and/or space-charge effects. Such behavior is physically plausible in the granular ZnO films, where grain boundaries, interfaces, and structural defects can provide localized electronic states that influence carrier transport. The change in slope across the switching region therefore suggests a modification of the available conduction pathways as the applied electric field increases.

A different behavior is observed for the TiO_2_ nanotubes (Figure 9b). The fitted slopes are approximately m=1.0 and m=1.6 before and after the switching region, respectively. Both values remain close to unity, indicating an approximately linear I-V dependence and therefore a predominantly Ohmic-like response over the analyzed voltage intervals. The small increase from 1.0 to 1.6 suggests that the switching event does not produce the pronounced transition toward strongly nonlinear transport observed for ZnO. Instead, the current remains compatible with conduction through pre-existing or electrically accessible pathways whose effective conductivity changes during resistive switching.

The comparison therefore reveals that the two oxide systems exhibit different bias-dependent transport signatures. ZnO shows a clear evolution from a weakly nonlinear regime m≈1.3 toward a nearly quadratic dependence (m≈1.8), whereas TiO_2_ remains close to the Ohmic limit m≈1.0 on both sides of the switching region. These results support the presence of a stronger trap- or defect-mediated contribution to the nonlinear conduction of ZnO, while transport in the analyzed TiO_2_ regions is comparatively more Ohmic-like. However, the power-law analysis alone does not uniquely identify the microscopic origin of resistive switching. In particular, it cannot by itself distinguish electronic trapping/detrapping from field-induced redistribution of oxygen-related defects. Consequently, these results are considered together with the temperature-dependent electrical measurements and structural and spectroscopic characterization when discussing the possible switching mechanisms.

The stability of the hysteretic response over a wide temperature range provides additional evidence supporting this mechanism. If the switching process were dominated by ionic migration, a significant suppression of the resistive switching behavior would be expected at cryogenic temperatures due to the drastic reduction of ionic mobility. However, both TiO_2_ nanotubes and ZnO thin films preserve well-defined hysteresis loops even down to low temperatures, indicating that charge transport remains governed by electronic processes. Although the current magnitude decreases as thermal activation is reduced, the persistence of the switching window demonstrates that the conductive pathways are sustained by defect-related electronic states that remain active under both vacuum and low-temperature conditions.

The different temperature dependence observed in TiO_2_ and ZnO can be attributed to their distinct microstructures. The vertically aligned nanotubular architecture of TiO_2_ provides quasi-one-dimensional conduction pathways along the nanotube walls, promoting the formation of stable defect-assisted electron channels. In contrast, ZnO thin films exhibit a granular morphology in which charge transport is additionally influenced by grain boundaries and surface states. Consequently, ZnO displays a stronger temperature dependence of the current magnitude, while TiO_2_ nanotubes exhibit greater robustness against thermal variations. Nevertheless, the preservation of hysteresis in both systems indicates that defect-mediated electronic conduction constitutes the dominant switching mechanism.

## 5. Conclusions

This work demonstrates that TiO_2_ nanotubes and ZnO thin films exhibit reproducible resistive-switching behavior over broad temperature ranges under high-vacuum conditions, although their electrical responses show different degrees of sensitivity to temperature. The results establish that resistive switching remains accessible in both oxide systems under the investigated environmental conditions and highlight the relevance of morphology, structural disorder, and defect-related electronic states in determining their electrical response. Within the experimental conditions investigated, TiO_2_ nanotubes exhibited comparatively greater cycling and thermal stability, whereas ZnO thin films showed a stronger temperature dependence of the current. Since the two systems differ in morphology, architecture, synthesis route, and investigated temperature range, this comparison is intended to establish qualitative trends rather than an intrinsic superiority of one material over the other. The main findings of this study can be summarized as follows. Raman spectroscopy, X-ray photoelectron spectroscopy, XRD measurements, and scanning electron microscopy confirmed the formation of defect-containing oxide nanostructures with distinct morphologies, consisting of self-aligned TiO_2_ nanotubes and granular ZnO thin films. Both materials exhibited stable hysteretic current–voltage characteristics associated with resistive switching, which were preserved under high-vacuum conditions and over a wide temperature range, demonstrating the robustness of the memory response against environmental perturbations. TiO_2_ nanotubes exhibited greater stability of the resistive states, maintaining an ON/OFF ratio close to 4.65 during endurance measurements, whereas ZnO thin films showed a stronger dependence of the current magnitude on temperature. Remarkably, the ZnO device exhibited a mean HRS/LRS resistance ratio of approximately 6.3, compared with 4.65 for the TiO_2_ nanotube device. Thus, ZnO provides a larger average resistance window under the investigated conditions, although its HRS shows greater cycle-to-cycle variability. In contrast, the TiO_2_ device exhibits a smaller resistance window but greater stability of both resistance states over the extended cycling test. The combined structural, spectroscopic, and electrical characterization indicates that the switching behavior is predominantly associated with defect-assisted electronic transport. In particular, oxygen vacancies and other defect-related electronic states can act as donor-like centers, contributing to charge-carrier generation and the formation of conductive percolation pathways between the electrodes. The persistence of hysteresis at cryogenic temperatures further supports the dominant role of electronic transport processes over long-range ionic migration. The comparative analysis also indicates that the nanotubular architecture of TiO_2_ provides greater thermal robustness than the granular ZnO morphology, favoring more stable conductive pathways and improved endurance of the resistive states. Overall, these findings highlight the suitability of both oxide nanostructures for non-volatile memory applications and provide insight into the role of defect-mediated electronic conduction in memristive devices operating under variable temperature and pressure conditions.

## Data Availability

Data sets generated during the current study are available from the corresponding author on reasonable request.

## Abbreviations

The following abbreviations are used in this manuscript:

XPS	X-ray Photoelectron Spectroscopy
SEM	Scanning Electron Microscopy
XRD	X-Ray Diffraction
TE	Top Electrode
